# Effects of Postharvest Time, Heat Treatment, pH and Filtration on the Limonin Content in Newhall Navel Orange (*Citrus sinensis* Osbeck cv. Newhall) Juice

**DOI:** 10.3390/molecules23102691

**Published:** 2018-10-19

**Authors:** Jun Zhang, Zhiqiang Yang, Yan Liang, Linyan Zhang, Wei Ling, Can Guo, Guangling Liang, Guotian Luo, Qin Ye, Balian Zhong

**Affiliations:** 1 National Engineering Research Center of Navel Orange, Gannan Normal University, Ganzhou 341000, China; 15707975909@163.com (Z.Y.); zjzzf2011@gmail.com (Y.L.); 18573488362@139.com (L.Z.); lw791010@163.com (W.L.); 15225960215@139.com (C.G.); 2 School of Chemistry and Chemical Engineering, Gannan Normal University, Ganzhou 341000, China; 15216101485@163.com (G.L.); guotianluo@gmail.com (G.L.); 3 Xinfeng Nongfu Spring Fruit Industry Co., Ltd, Ganzhou 341000, China; qye02@mail.nfsq.com.cn

**Keywords:** delayed bitterness, limonin, navel orange, Newhall

## Abstract

Delayed bitterness causes severe economic loss in citrus juice industry worldwide, which is mostly due to the formation of limonoid compounds, especially limonin, in juice. In this study, effects of postharvest time of fruits, heat treatment, pH and filtration of juice on limonin content in Newhall navel orange (*Citrus sinensis* Osbeck cv. Newhall) juice were investigated. Our research indicated for the first time that: (1) limonin content in juice would gradually increase to a maximal level and then remained almost constant thereafter as storage time going on, whereas the maximum constant value (MCV) of limonin content in juice significantly (*p* < 0.05) decreased with the increment of postharvest time of fruits being juiced; (2) heat treatment and acidification of juice only speeded up the formation of limonin to the maximal level while without changing the MCV of limonin content; (3) the juice after filtration exhibited much lower MCV of limonin content compared with the unfiltered one. These experimental observations might not only provide useful information for the development of new debitterness method for navel orange juice, but also strongly support the acid-promoted delayed bitterness mechanism, suggesting the formation of delayed bitterness might primary due to the acid-promoted rather than the enzyme-catalyzed lactonization of limonoate A-ring lactone (LARL) to produce limonin in juice of navel orange.

## 1. Introduction

**Figure 1:**
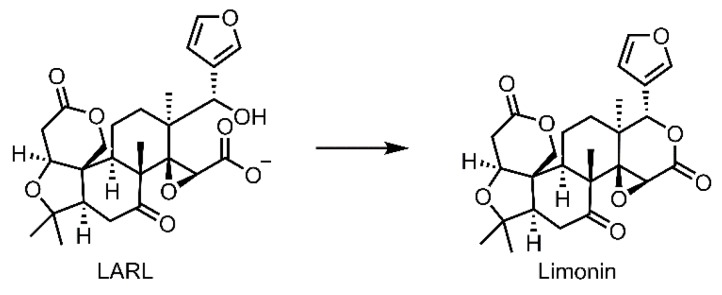
Conversion of non-bitter precursor LARL to bitter compound limonin.

Due to its pleasant sensory and healthy beneficial properties, orange juice attracts the largest number of juice consumers worldwide [1]. Delayed bitterness is one of the major problems facing the citrus juice industry which is characterized by gradual formation of bitter compounds in juice on standing after its extraction from the fruit which is normally non-bitter when eaten fresh [2]. Limonin, an intensely bitter triterpenoid dilactone (Figure 1), was identified as the main compound responsible for the delayed bitterness in navel orange juice [3,4]. During the past years, despite tremendous efforts, the mechanism underlying delayed bitterness has remained kind of unclear and controversial. Kefford put forth the diffusion theory where he suggested that limonin itself was present in the fruit tissues, but because of its low solubility it took an appreciable time to diffuse from the tissue fragments into juice [5]. Based on thin layer chromatography (TLC) and paper electrophoretic analyses, Maier suggested that a non-bitter limonin precursor, limonoate A-ring lactone (LARL) instead of limonin itself, was present in the albedo and endocarp tissues, and was released from the tissues when fruit was juiced and then came in direct contact with the acidic juice to form the bitter compound limonin [6]. However, the chemical basis of this acid-elicited delayed bitterness mechanism was obscure, lacking some systematic studies and direct evidences. Followed the identification of limonoid D-ring lactone hydrolase, an enzyme that could catalyze the lactonization of open D-ring from the citrus seeds [7], the formation of limonin from LARL was then arbitrarily believed to proceed under acidic conditions and aided by this enzyme in citrus juice [8,9,10]. Notably, Hasegawa pointed out the activity of limonoid D-ring lactone hydrolase, which catalyzed the lactonization of the open D-ring, occurred only in the seeds of citrus [7,11]. For the juice from Newhall navel orange which normally has no seed [12], whether the delayed bitterness was related to this enzyme remained unknown. 

Newhall (*Citrus sinensis* Osbeck cv. Newhall), is a citrus fruit cultivar widely planted in Ganzhou, located in the south of Jiangxi Province, China. In part due to the special climate conditions and the rare earth-abundant cultivation soils in Ganzhou, the Newhall oranges produced here enjoy plenty of excellent attributes, including s beautiful appearance, appreciated flavor, pleasant color, and so on. Currently, the navel orange produced in Ganzhou, briefly named Gannan navel orange, is a national geographical indication product with ‘*Gannan navel orange*’ being a Chinese well-known trademark [13]. By the end of 2017, the total planting areas of navel orange (over 90% being Newhall) in Ganzhou had reached around 103.2 thousand hectares, with an annual output of about 1.24 million tons [14], making Ganzhou the largest navel orange production area in China. In contrast to other main orange-producing countries like Brazil and USA where most of the oranges are processed into juices, over 90% of Gannan navel oranges were consumed by being freshly eaten. This sole ‘freshly eaten’ consumption method has seriously limited the further development of the navel orange planting industry in Ganzhou. Thus, it was very urgent to explore multiple consumption methods for Gannan navel orange. Juicing is one of the most popular and value-added fruit processing methods. However, one of the main problems encountered during navel orange juice processing is the phenomenon of delayed bitterness. Over the past decades, navel oranges have been continuously considered to be unsuitable for juice processing due to a severe delayed bitterness phenomenon, especially for the early to mid-season fruits [15,16]. Therefore, searching for efficient and practical debittering methods is very important for juice production from Gannan navel oranges.

Several debittering methods have been introduced to reduce the contents of bitter compounds in citrus juices, typically involving, but not limited to, the addition of bitterness suppressing agents [17,18], biodegradation by enzymes [19,20], film filtration [21], and adsorption on certain adsorbents [22,23]. The most commonly used method to reduce the bitterness in processed citrus juices was the use of polymeric adsorbents to selectively remove limonoids. However, in addition to the bitter compounds, some beneficial compounds such as vitamin C, carotenoids, or phenolic compounds might be reduced as well by this debittering method [24,25,26]. Although they reduce the bitterness to some extent, the conventional debittering methods, such as the addition of bitterness-suppressing agents, or by adsorption on certain adsorbents, all cause more or less negative effects on the quality of the juices [1,17,21,22], precluding their practical application in the juice industry. As a result, the identification of novel debittering methods which can efficiently reduce the bitterness while keeping the maximum natural nutrition and original taste of juice, was of great interest to the researchers in the citrus juice production field.

During our efforts to overcome the bottleneck in juice production from Gannan navel orange, the delayed bitterness, our research group has initiated a series of research works. In this study, using HPLC analysis, we investigated whether the postharvest time of fruits, the heat treatment, pH, and the insoluble membrane tissues of juice, have any effect on the limonin content in juice, aiming to identify potential debittering methods and shed some light on the mechanism underlying the delayed bitterness of juice extracted from navel oranges.

## 2. Results and Discussion

### 2.1. Limonin Extraction and Its Measurement by HPLC

A robust, stable and effective method of extracting limonin from juice samples is essential for monitoring the limonin content in juice. After careful comparisons of the extracting efficiency, operating convenience, and reliability by using various extracting solvents, dichloromethane (DCM) was chose as extraction solvent due to its high density relative to water, excellent solubility toward limonin and relatively low boiling point, enabling the clear separation of two layers in the separating funnel and easy removal of solvent in the concentration step. The limonin extraction method employed in our experiment was believed to be stable and efficient as revealed by the standard addition experiment which displayed good extraction recovery percentage of 98.6 ± 2.1%. The calibration curve of a limonin standard was constructed by plotting the peak areas versus the concentrations of the standard, which showed a correlation coefficient of 0.9996. The LOD and LOQ of the HPLC method were 0.32 and 0.62 mg/L, respectively. Precision tests of the method demonstrated that the intra-day and inter-day RSDs (%) were 0.40% and 0.98%, respectively. These results indicated that our method for the extraction and measurement of limonin from juice was accurate and reliable.

### 2.2. Effect of Postharvest Time on the Limonin Content in Juice

**Figure 2:**
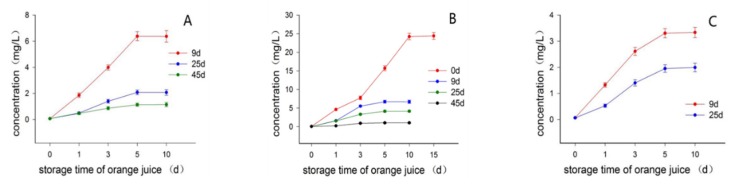
Effect of postharvest time on the limonin content in juice. (**A**) Batch one fruits were used; (**B**) Batch two fruits were used; (**C**) Batch three fruits were used.

Three batches of fruits collected in Nov 14 (batch 1), 29 (batch 2) and Dec 12 (batch 3) 2017, respectively, were used in this experiment. The limonin content in freshly squeezed juice (day 0 of storage time) was very low, ranging from 0.06 to 0.07 mg/L, for all three batches of fruits (Table S1). As juice storage time went on, the limonin content gradually increased to a maximal level and then remained almost constant in juice, as shown in Figure 2. For the fruits of batch 1 (Figure 2A), the MCV of limonin content in the juices extracted from the fruits after 9, 25, 45 d of postharvest time, were around 6.37, 2.07, and 1.13 mg/L, respectively, which were significantly (*p* < 0.05) different from each other, clearly indicating that the postharvest time of fruits significantly influenced the MCV of limonin content in juices which obviously reduced with the increment of the postharvest time of fruits (Figure 2A). This conclusion was also supported by the experimental data shown in Figure 2B,C. For the fruits of batch 2 (Figure 2B), the maximal level of limonin content in juice from freshly collected fruits (day 0 of postharvest time) was reached after 10 d of juice storage time with the MCV being around 24.24 mg/L, which was much higher than those of juices extracted from the fruits 9, 25 and 45 d after postharvest time, being around 6.68, 4.15 and 1.04 mg/L, respectively (Table S1), supporting the conclusion that the MCV of limonin content in juice decreased with the increment of postharvest time of fruits being juiced. The experimental result (Figure 2C), obtained from the batch 3 fruits, was in good agreement with those of batches 1/2, with the MCV being around 3.34 and 1.99 mg/L in the juices extracted from the fruits after 9 and 25 d postharvest time, respectively. Consequently, based on the consistent experimental data obtained from these three independent batches of fruits, it was reasonable to conclude that the limonin content would gradually increase to a maximal level in juice on standing, whereas the MCV was highly influenced by the postharvest time of fruits being juiced, which significantly decreased with the increment of postharvest time. In view of the experimental result, we tentatively assumed that the LARL, a precursor of limonin, would convert to limonin 17-β-d-glucoppanoside (LG) in the fruit tissues by the limonoate dehydrogenase during the postharvest time, leading to much lower MCV of limonin content in juice, which was similar to the procedure reported previously that the LARL would gradually convert to LG during the fruit growth and maturation [27]. This experimental result might provide a convenient and practical debittering application for navel orange juices by juicing the fruits stored for an appropriate time instead of freshly collected ones. It was worth noting that this proposed potential debittering application might represent a completely natural way without any addition of artificial substances, maximizing the original taste of juice, which might exhibit tremendous application potential in the manufacture of navel orange juice. The appropriate postharvest time considering both debittering and other chemico-physical properties of fruits remains to be determined.

### 2.3. Effect of Heat Treatment on the Limonin Content in Juice

**Figure 3:**
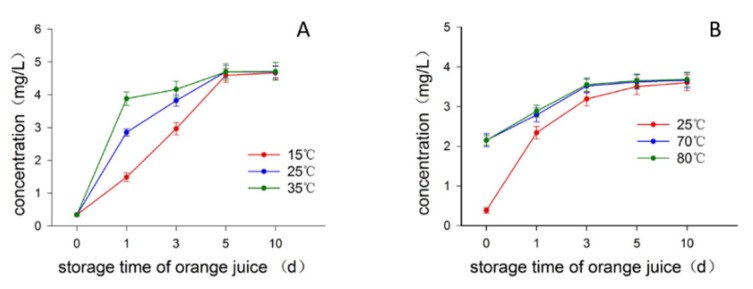
Effect of heat treatment on the limonin content in juice. (**A**) Treated at 15, 25 and 35 °C respectively; (**B**) Treated at 25, 70 (lasted ten min) and 80 °C (lasted ten min), respectively.

Since the most commonly used disinfection method for the production of juice is pasteurization, we investigated how the heat treatment of juice influenced the formation of limonin in juice on standing. The batch 2 fruits with 45, 55 d postharvest time were used in this part. Figure 3A displayed the change of limonin content in juices from the fruits of 45 d postharvest time when the juices were statically stored at three different temperatures of 15, 25, and 35 °C, respectively. After one day of storage time, the limonin content in the juice stored at 35 °C sharply increased to around 3.88 mg/L, followed by the juice stored at 25 °C being around 2.86 mg/L, while only a small increase in the juice stored at 15 °C (around 1.49 mg/L), indicating the high temperature greatly speeded up the formation of limonin. However, as time goes on, the difference of limonin content caused by the different heat treatments among these juices gradually decreased, and the limonin content in all juices stored at different temperatures reached to almost the same value at day 5 of storage time, and kept almost stable thereafter as revealed by limonin contents at days 5 and 10 of the storage time, being almost the same with MCV of around 4.65~4.71 mg/L (Table S2). Consistent with the observation from the fruits of 45 d postharvest time, the data obtained from the fruits of 55 d postharvest time revealed almost the same trend in response to the heat treatment (Figure 3B). The limonin content quickly reached around 2.15 mg/L for the juices subjected to short-time storage while high-temperature treatments (ten min at 70 or 80 °C, then cooled down and stored at 25 °C) at the treatment day (day 0 of juice storage time) (Table S2), significantly higher than that of juices constantly stored at 25 °C (0.38 mg/L), then as storage time going on, the limonin content of all juices increased and reached to almost the same MCV being around 3.60–3.68 mg/L (Table S2). Interestingly, no matter what heat treatments conducted, the MCVs of limonin content in juices from the fruits of same postharvest time were almost the same, indicating that the LARL content was stable and constant for the fruits of same harvest and postharvest time, independent of the heat treatment. Provided there were constant amount of LARL in the tissues of fruit, it could be assumed that, as its conversion to limonin, the LARL amount left would decrease, consequently reducing the formation speed of limonin. This assumption was supported by the experimental observation, as depicted in Figure 3, where the increment speeds of limonin in all juice samples were much high in the first few days of storage time while gradual slowing down as the storage time going on. Therefore, as demonstrated in Figure 3 and Table S2, it could be deduced that the heat treatment of juices only speeded up the formation of limonin while without changing the MCV of limonin content in juice. This observation prompted us to suspect the involvement of the enzyme limonin D-ring lactone hydrolase in limonin formation, because normally the enzyme would be inactivated in high temperature [7]. Then we carried out experiments to determine whether the formation of limonin in juice was acid-promoted or enzyme-catalyzed procedure by investigating the effect of pH on the limonin content in juice.

### 2.4. Effect of pH on the Limonin Content in Juice

**Figure 4:**
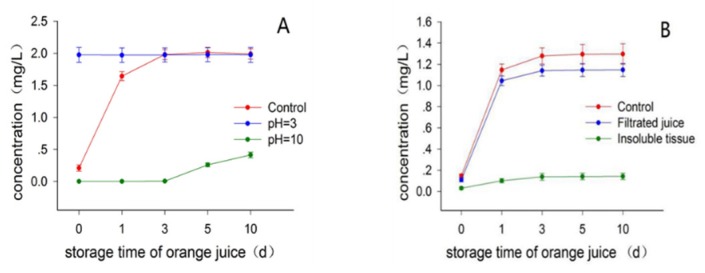
Effects of pH (**A**) and filtration (**B**) on the limonin content in juice.

Batch 3 fruits with 50 d postharvest time were used in this experiment. To investigate whether the pH of juice had some effect on the limonin formation, aqueous acid or base was intentionally added into the juice with no-added juice as control. As shown in Figure 4A, the limonin content in the acidified juice (pH 3) quickly increased to the maximal level upon the addition of acid, being around 1.98 mg/L (day 0 of storage time), sharply comparing to the control juice being around 0.21 mg/L, suggesting that the acid significantly promoted the formation of limonin. Interestingly, during the following storage time, the limonin content in the acidified juice almost kept unchanged, being around 1.97~1.98 mg/L, while the limonin content in the control juice gradually increased and almost reached to the same maximal level as that of the acidified juice at day 3 of storage time, then kept stable with MCV around 1.99~2.01 mg/L. These data suggested that the acid addition, being consistent with the heat treatment, could significantly accelerate the limonin formation, while causing no influence on the MCV of juice on standing. This suggestion was further supported by the base-added experiment. There was no detectable limonin in the base-added juice (pH 10) during the first three days of storage time, and only limited amount of limonin (around 0.26 mg/L) was formed until the day 5 of storage time, which was slowly increase to around 0.41 mg/L after stored for ten days (Table S3). A plausible explanation for the limited amount of limonin formation in the basified juice after long-time storage, was that the base might be consumed as time going on, by some other compounds such as varieties of esters in juice, bringing back a neutral or weak acid condition in the juice, then promoting the slow formation of limonin. Considering these experimental observations, we assumed that the formation of limonin from the precursor LARL in navel orange juice, should be an acid-promoted reaction which was accelerated by the addition of acid and heat treatment, presumably excluding the involvement of limonoid D-ring lactone hydrolase. In view of the fact that navel orange normally has no seed [12], this assumption was supported as well by the previous literatures where reported that the limonoid D-ring lactone hydrolase was only present in the seeds of citrus [7,8,11].

### 2.5. Effect of Filtration on the Limonin Content in Juice

Batch 3 fruits with 60 d of postharvest time were used in this experiment. Maier suggested that the limonin precursor LARL might be present in the albedo and endocarp tissues of the fruit [6], which inspired us to examine whether the removal of the insoluble tissues from juice by filtration would have some effects on the limonin formation in juice. In good agreement with our expectation, the filtration of the insoluble tissue obviously reduced the limonin formation in juice, as shown in Figure 4B. There were significantly different levels of limonin concentration between the filtered and the control juice during the whole storage time of juice (days 0 to 10), with the MCV being around 1.15, 1.30 mg/L for the filtrated and control juices (Table S3), respectively. Interestingly, limonin was also detected in the suspension of the insoluble tissue of juice in acidified aqueous, which gradually increased to a maximal level with the MCV being around 0.14 mg/L. Interestingly, when the MCVs of the limonin content in both the filtered juice (1.15 mg/L) and the insoluble tissues (0.14 mg/L), was added up, the obtained total MCV (1.29 mg/L) was almost the same as that of unfiltered control juice (1.30 mg/L). These data support the suggestion that the limonin precursor LARL should be present in the membrane tissue of the fruit, and quickly passed into the juice when the fruit was juiced, explaining the experimental observation of limonin reduction with the removal of the membrane tissue in juice. Due to the very quickly release of LARL into juice from the membrane tissue, there was still a large amount of limonin formed in the filtered juice on standing, even though the immediate removal process was conducted. This experimental result presumably demonstrated a practical debittering application for navel orange juice by immediate removal of the insoluble tissues from the juice obtained. Notably, this potential debittering method is a physical procedure, it could reduce the bitterness without any addition of external artifacts, keeping the juice natural and safe. Moreover, this potential debittering method would produce a clear orange juice which might be preferred by some consumers.

## 3. Materials and Methods

### 3.1. Chemicals

Limonin was obtained commercially (Shanghai Yuanye Bio-Technology Co., Ltd., Shanghai, China). Potassium sorbate (Shanghai Linfeng Co., Putuo, Shanghai, China), and dichloromethane were of analytical grade (Damao Chemical Reagent Factory, Tianjin, China), while methanol and acetonitrile were HPLC grade (Anaqua Chemicals Supply, Houston, TX, USA), and de-ionized water used for chromatography was obtained from a Milli-Q Gradient A10 system (Millipore, Billerica, MA, USA).

### 3.2. Fruits Materials

Newhall navel oranges were harvested from an orchard at the National Engineering Research Center of Navel Orange located in Ganzhou City, in South China’s Jiangxi province. Three healthy citrus trees growing under the same climate, weather, and farming conditions, were selected for samples collection. Three batches of fruits at commercial mature stage were collected from these three trees selected on November 14 (batch 1), November 29 (batch 2), and December 12 (batch 3) 2017, respectively. Each batch of fruit contained 150 fruits (50 from each tree) collected from the different parts of crown with similar size and normal color. All fruits were send to laboratory and stored at ambient conditions (temperature 20–25 °C & relative humidity 70–90%) until analysis. Fruit was cut in half using domestic kitchen knife, which then was juiced using a domestic semi-automatic juicer (Mode 4161, Braun, De’Longhi Braun Household GmbH, Budapest, Hungary).

### 3.3. Effect of Postharvest Time on the Limonin Content in Juice

On the days 9, 25, and 45 of postharvest time, five fruits from batch 1 were taken for juice extraction, respectively. Each time, the juice obtained (~0.4 L) was blended with potassium sorbate (0.1% *wt*/*v*) to form a homogenous suspension, which was then transferred to the centrifuge tubes (Eppendorf, Hamburg, Germany, 50 mL), each tube containing 20 mL juice, with 15 tubes in total. Then the tubes were lidded and stored statically at 25 °C. On the days 0, 1, 3, 5, 10 of storage time of juices, three tubes were randomly taken out for the analysis of limonin content, respectively. For the fruits from batch two, in addition to the same procedure as that of fruits from batch one, the limonin content in the juice extracted from the freshly collected fruits (day 0 of postharvest time) was analyzed on the days 0, 1, 3, 5, 10, 15 of storage time of juices. For the fruits from batch three, the limonin content was analyzed according to the same procedure as that of fruits from batch one, whereas only the days 9 and 25 of postharvest time were investigated. The data reported in this study were the average of three tubes.

### 3.4. Effect of Heat Treatment on the Limonin Content in Juice

Batch 2 fruits were used in this experiment. On day 45 of postharvest time, 12 fruits were taken for juice extraction. The juice obtained (~1.0 L) was blended with potassium sorbate (0.1% *wt*/*v*) to form a homogenous suspension, which was then transferred to the centrifuge tubes, each tube containing 20 mL juice, with 45 tubes in total. Then the tubes were lidded and randomly separated into three sets (15 tubes each set), which were stored statically at 15, 25 and 35 °C. On the days 0, 1, 3, 5, 10 of the storage time of juices, three tubes were randomly taken out from each set for the analysis of limonin content, respectively. On the day 55 of postharvest time, 12 fruits were taken out for juice extraction. The juice obtained (~1.0 L) was processed using the same method aforementioned, while being subjected to different heat treatment. The tubes were lidded and randomly separated into three sets (15 tubes each set), one set was stored statically at 25 °C, and another two sets were heated for ten min at 70 and 80 °C, respectively, then cooled down and stored statically at 25 °C. On the days 0, 1, 3, 5, 10 of the storage time of juices, three tubes were randomly taken out from each set for the analysis of limonin content, respectively.

### 3.5. Effect of pH on the Limonin Content in Juice

Batch 3 fruits were used in this part. Sixteen fruits with 50 d postharvest time were taken out for juice extraction. The juice obtained (~1.3 L) was blended with potassium sorbate (0.1% *wt*/*v*) to form a homogenous suspension, which was then evenly separated into three sets (0.4 L each set). One set was directly transferred to the centrifuge tubes, each tube containing 20 mL juice, with 15 tubes in total. The other two sets were adjusted to pH 3 and 10, respectively, by addition of acetic acid (22 mL), or 1 M NaOH aqueous (25 mL), which were then transferred to the centrifuge tubes, each tube containing 20 mL juice sample, with 15 tubes in total for each set. Then the tubes of all three sets were lidded and stored statically at 25 °C. On the days 0, 1, 3, 5, 10 of the storage time of juices, three tubes were randomly taken out from each set for the analysis of limonin content, respectively. For the calculation of the last two sets with acid or base being added, the actual juice sample volumes of 19 mL (acid-added set), and 18.8 mL (base-added set) were used instead of 20 mL in each tube.

### 3.6. Effect of Filtration on the Limonin Content in Juice

Batch 3 fruits with 60 d postharvest time were used in this experiment. Eleven fruits were taken out for juice extraction. The juice obtained (~0.9 L) was blended with potassium sorbate (0.1% *wt*/*v*) to form a homogenous suspension, which was then separated into two sets. One set (0.3 L) was transferred to the centrifuge tubes, each tube containing 20 mL juice, with 15 tubes in total. The other set (0.5 L) was subjected to centrifugation (8000 rmp, 8 min, Eppendorf), then the supernatant was transferred to the centrifuge tubes, each tube containing 20 mL juice, with 15 tubes in total. The insoluble tissue fragments of the juice, obtained after centrifugation, were transferred to a beaker containing 0.4 L CH_3_COOH aqueous (pH 3) and blended to form a homogenous suspension, which was then transferred to the centrifuge tubes, each tube containing 20 mL, with 15 tubes in total. Then the tubes of all three sets were lidded and stored statically at 25 °C. On the days 0, 1, 3, 5, 10 of storage time of juices, three tubes were randomly taken out from each set for the analyses of limonin content, respectively. Since only 18.5 mL of supernatant was obtained from 20 mL juice, therefore, for the calculation of limonin content in the first set of juices, the actual juice sample volume of 18.5 mL was used instead of 20 mL for in parallel comparisons.

### 3.7. Limonin Extraction from Juice Samples

De-ionized water (10 mL) was added into the tube contained juice sample (20 mL) to form a diluted suspension, which was transferred to a separating funnel. The tube was then washed by de-ionized water (5 mL × 2) to make sure all the samples was transferred into the funnel. The juice suspension was then extracted with dichloromethane (40 mL × 3). The combined dichloromethane solution was concentrated by rotary evaporator under reduced pressure at 50 °C to furnish a residue, which was dissolved with 1 mL methanol. The methanol solution was then filtrated through a 0.22 μm size hydrophilic polytetrafluoroethylene (PTFE) syringe filter into the HPLC vial (2 mL, Agilent Technologies, Palo Alto, CA, USA), followed by washing the flask and filter (~0.2 mL × 2) with methanol, making sure the final total volume of the methanol solution in the vial was exactly fixed at 1.5 mL. The recovery ratio of the extraction method was determined by adding certain amount of limonin standard to the juice sample in which the limonin concentration has been measured, followed by extraction using the aforementioned method. Three replicates by adding different amount of limonin standard were carried out for each concentration.

### 3.8. Measurement of Limonin by HPLC

For the chromatographic measurement of limonin, an Agilent Technologies 1220 Infinity system which consisted of a UV detector, a binary gradient pump, and a Sunfire ™C18 reverse-phase column (150 mm × 4.6 mm id, 3.5 μm particle size) was employed. The mobile phase consisted of acetonitrile (A) and de-ionized water (B) at a flow rate of 1.0 mL/min. The gradient profile was as follows: 0–30 min, 10–70% A; 30–40 min, 70–90% A; 40–50 min, 90% A; 50–60 min, 10% A. The chromatogram was monitored at 210 nm with the injection volume fixed at 20 μL and column temperature kept at 30 °C. Limonin was identified by comparisons of its retention time and UV spectrum with those of the standard and the concentration was quantified by external calibration method. Since the crude extract of all juice samples were finally fixed at constant volume of 1.5 mL in HPLC vial, the original limonin concentration in the juice samples should be calculated by the following equation:

(1) $$Original\;limonin\;concentration=\frac{Concentration\;from\;the\;calibration\;curve\times 1.5}{Volume\;of\;sample}$$

### 3.9. Establishment of External Standard Curve and Validation of the HPLC Method

Seven concentrations (0.5, 1.0, 2.0, 4.0, 8.0, 15.0, 30.0 mg·L^−1^) of limonin standard were prepared and stored at 0–4 °C in refrigerator, which were then measured by HPLC using the methods and conditions aforementioned. The calibration curve was constructed by plotting the peak areas versus the concentrations of the limonin standard. Validations of the HPLC method were based on limit of detection (LOD), limit of quantification (LOQ), and precision. The LOD was evaluated by decreasing concentrations of limonin standard until a response equivalent to three times the background noise was observed, while the LOQ was evaluated by the ratio of signal-to-noise of at 10. The relative standard deviation (RSD) was taken as a measure of precision. Intra-day and inter-day repeatability were evaluated by measuring the limonin standard concentrations on six replicates within one day and three consecutive days, respectively.

### 3.10. Statistical Analysis

Data were expressed as means ± standard deviation of three replicates. Analysis of one way variance (ANOVA) was used to compare the means, and determine significant differences (*p* < 0.05). All statistical analyses were processed by the DPS software (Version 11.5, Refine Information Tech. Co., Ltd. Hangzhou, China).

## 4. Conclusions

In our study, we investigated the effects of postharvest time of fruits, heat treatment, pH, and filtration of juice on limonin content in Newhall navel orange (*Citrus sinensis* Osbeck cv. Newhall) juice. The results revealed here might not only provide useful information for the development of novel debittering methods for the manufacture of juice from navel oranges such as by juicing the fruits stored for an appropriate time instead of freshly collected ones, and by immediate removal of some insoluble tissues from the juice obtained, but also might shed substantial light on the delayed bitterness mechanism of navel orange juice, suggesting the formation of delayed bitterness might mainly due to the acid-promoted rather than the enzyme-catalyzed lactonization of LARL to produce limonin in juice of navel orange.

## Supplementary Materials

The following are available online. Table S1: Effect of postharvest time on the limonin content (mg/L) in juice; Table S2: Effect of heat treatment on the limonin content (mg/L) in juice; Table S3: Effects of pH and filtration on the limonin content (mg/L) in juice.
