# Supplementary material for: Effects of Postharvest Time, Heat Treatment, pH and Filtration on the Limonin Content in Newhall Navel Orange (Citrus sinensis Osbeck cv. Newhall) Juice
Source: Molecules. 2018 Oct 19;23(10):2691. doi: 10.3390/molecules23102691 (PMC6222338; doi:10.3390/molecules23102691)
Supplement: Supplementary file 1 [file molecules-23-02691-s001.pdf]

Effects of postharvest time, heat treatment, pH and filtration on the limonin content in Newhall navel orange (*Citrus sinensis* Osbeck cv. Newhall) juice

Jun Zhang [1,*], Zhiqiang Yang [1], Yan Liang [1], Linyan Zhang [1], Wei Ling [1], Can Guo [1], Guangling Liang [2], Guotian Luo [2], Qin Ye [3], Balian Zhong [1,*]

[1] National Engineering Research Center of Navel Orange, Gannan Normal University, Ganzhou, 341000, China; 15707975909@163.com (Z.Y.); zjzzf2011@gmail.com (Y.L.); 18573488362@139.com (L.Z.); lw791010@163.com (W.L.); 15225960215@139.com (C.G.)

[2] School of Chemistry and Chemical Engineering, Gannan Normal University, Ganzhou, 341000, China; 15216101485@163.com (G.L.); guotianluo@gmail.com (G.Luo)

[3] Xinfeng Nongfu Spring Fruit Industry Co., Ltd, Ganzhou, 341000, China; qye02@mail.nfsq.com.cn (Q.Y.)

* Correspondence: bri71527152@outlook.com (J.Z.); bal.zh@163.com (B.Z.); Tel.: +86-797-839-3068 (J.Z.)

Table S1. Effect of postharvest time on the limonin content (mg/L) in juice.

	Batch one fruits			Batch two fruits				Batch three fruits	
PTF [a] (d) / STJ [b] (d)	9	25	45	0	9	25	45	9	25
0	0.07Da（±0.02）	0.07Da（±0.01）	0.07Ca（±0.02）	0.07Ea（±0.02）	0.06Da（±0.01）	0.06Da（±0.02）	0.06Ba（±0.01）	0.07Da（±0.01）	0.06Da（±0.01）
1	1.86Ca（±0.18）	0.50Cb（±0.08）	0.47Bb（±0.08）	4.62Da（±0.22）	1.59Cb（±0.13）	1.53Cb（±0.08）	0.22Bc（±0.04）	1.33Ca（±0.09）	0.53Cb（±0.06）
3	3.99Ba（±0.19）	1.40Bb（±0.14）	0.87Ac（±0.12）	7.73Ca（±0.49）	5.52Bb（±0.20）	3.31Bc（±0.20）	0.90Ad（±0.09）	2.61Ba（±0.15）	1.40Bb（±0.12）
5	6.38Aa（±0.35）	2.08Ab（±0.18）	1.13Ac（±0.12）	15.72Ba（±0.66）	6.70Ab（±0.40）	4.14Ac（±0.25）	1.02Ad（±0.12）	3.31Aa（±0.18）	1.95Ab（±0.15）
10	6.37Aa（±0.44）	2.07Ab（±0.22）	1.14Ac（±0.16）	24.24Aa（±0.88）	6.68Ab（±0.44）	4.15Ac（±0.25）	1.04Ad（±0.14）	3.34Aa（±0.20）	1.99Ab（±0.17）
15	—	—	—	24.42A（±0.90）	—	—	—	—	—

a: Postharvest Time of Fruits; b: Storage Time of Juices; —: Not applicable; The data were reported as average ± SD (three replicates); The different lowercase letter in the same line within the same batch of fruits indicated significant difference ($p < 0.05$), the different uppercase letter in the same column within the same batch of fruits indicated significant difference ($p < 0.05$).

Table S2. Effect of heat treatment on the limonin content (mg/L) in juice.

	Fruits with 45d of postharvest time			Fruits with 55d of postharvest time		
HTJ [a] / STJ [b] (d)	15℃	25℃	35℃	25℃	70℃ [c]	80℃ [c]
0	0.35Da（±0.05）	0.34Da（±0.05）	0.34Ca（±0.04）	0.38Db（±0.07）	2.15Ca（±0.17）	2.15Ca（±0.13）
1	1.49Cc（±0.14）	2.86Cb（±0.11）	3.88Ba（±0.21）	2.34Cb（±0.16）	2.79Ba（±0.18）	2.89Ba（±0.15）
3	2.97Bb（±0.19）	3.82Ba（±0.17）	4.16Ba（±0.25）	3.19Bb（±0.18）	3.52Aab（±0.17）	3.55Aa（±0.17）
5	4.65Aa（±0.20）	4.70Aa（±0.20）	4.70Aa（±0.25）	3.51Aa（±0.21）	3.62Aa（±0.18）	3.65Aa（±0.17）
10	4.67Aa（±0.20）	4.70Aa（±0.18）	4.71Aa（±0.27）	3.60Aa（±0.20）	3.66Aa（±0.19）	3.68Aa（±0.18）

a: Heat Temperatures of Juices; b: Storage Time of Juices; c: The heat treatment lasted only ten minutes, then cooled down and stored at 25℃; The different lowercase letter in the same line within the same postharvest time of fruits indicated significant difference ($p < 0.05$), the different uppercase letter in the same column within the same postharvest time of fruits indicated significant difference ($p < 0.05$). The data were reported as average ± SD (three replicates).

Table S3. Effects of pH and filtration on the limonin content (mg/L) in juice.

	Effect of pH of juice [a]			Effect of filtration of juice [b]		
TMJ [c] / STJ [d] (d)	Control	pH=3	pH=10	Control	Filtrated juice	Insoluble tissue (pH=3)
0	0.21Cb (±0.05)	1.98Aa (±0.12)	ND [e]	0.15Ca (±0.02)	0.11Cb (±0.02)	0.03Bc (±0.01)
1	1.64Bb (±0.07)	1.97Aa (±0.11)	ND [e]	1.15Ba (±0.06)	1.05Bb (±0.05)	0.10Ac (±0.02)
3	1.98Aa (±0.08)	1.97Aa (±0.11)	ND [e]	1.28ABa (±0.08)	1.14Ab (±0.05)	0.14Ac (±0.03)
5	2.01Aa (±0.08)	1.98Aa (±0.11)	0.26Bb (±0.03)	1.30Aa (±0.09)	1.15Ab (±0.06)	0.14Ac (±0.03)
10	1.99Aa (±0.08)	1.98Aa (±0.12)	0.41Ab (±0.04)	1.30Aa (±0.10)	1.15Ab (±0.06)	0.14Ac (±0.03)

a: Batch three fruits with 50d of postharvest time were used; b: Batch three fruits with 60d of postharvest time were used; c: Treatment methods of juices; d: Storage time of juices; The different lowercase letter in the same line within the same postharvest time of fruits indicated significant difference ($p < 0.05$), the different uppercase letter in the same column within the same postharvest time of fruits indicated significant difference ($p < 0.05$); e: Not Detected; The data were reported as average ± SD (three replicates).
